# Generation of NADPH and the formation of lipofuscin fluorophore precursors in human rod photoreceptors

**DOI:** 10.1016/j.jbc.2025.110802

**Published:** 2025-10-08

**Authors:** Leopold Adler, Chunhe Chen, Nicholas P. Boyer, Yiannis Koutalos

**Affiliations:** Department of Ophthalmology, Medical University of South Carolina, Charleston, South Carolina, USA

**Keywords:** fluorescence, human retina, lipofuscin, metabolism, photoreceptor, vision, vitamin A

## Abstract

With photoreceptor cell death being one of the major causes of vision loss, we undertook a study of photoreceptor metabolic competence by measuring the generation of NADPH, which is used in synthetic reactions and the reduction of all-*trans* retinal to all-*trans* retinol. All-*trans* retinal is released within rod photoreceptors during light detection and can form lipofuscin fluorophore precursors, which accumulate in the form of the cytotoxic pigment lipofuscin in the adjacent cells of the retinal pigment epithelium. We have used fluorescence imaging to measure the levels of all-*trans* retinal, all-*trans* retinol, and lipofuscin fluorophore precursors in single living rod photoreceptors isolated from human donor eyes. We supplied isolated rods with exogenous all-*trans* retinal and used its reduction to all-*trans* retinol to measure their capacity to generate NADPH. Although the exogenous all-*trans* retinal loads were much higher than those of the endogenous released during light detection, the cells were able to sustain the reduction of a steady 80 to 90% proportion to all-*trans* retinol, similar to that maintained for the reduction of the endogenously generated. Generation of NADPH required the presence of extracellular glucose. The capacity to generate NADPH deteriorated with time after donor death and isolation of the retina, resulting in increased formation of lipofuscin fluorophore precursors by the supplied all-*trans* retinal. This suggests that in the living eye, an initial deterioration of rod photoreceptor metabolism would result in increased lipofuscin accumulation and impairment of retinal pigment epithelium function, which would lead to further deterioration of photoreceptor cell health and eventual death.

Cells rely on robust metabolism to meet the energy demands of their physiological functions and maintain their viability. In the case of rod photoreceptor cells, a robust metabolism is necessary to sustain the high energy requirements of light detection (1), as well as the continuous renewal of the outer segment (2), the compartment that contains the light-detecting machinery. Rod outer segment renewal is supported by synthetic reactions that depend on the generation of large amounts of NADPH (3, 4). In addition to supporting the synthetic reactions, NADPH is also used in the recycling of the chromophore of the visual pigment rhodopsin, which is destroyed during light detection. Light isomerizes the 11-*cis* retinyl chromophore of rhodopsin to all-*trans*, forming an active form that initiates the reactions that culminate in a change in rod cell membrane potential, thereby accomplishing the conversion of light to an electrical signal (5). Recycling of the photoisomerized retinyl chromophore begins with its release in the form of all-*trans* retinal by photoactivated rhodopsin. The released all-*trans* retinal is reduced within the rod outer segment by retinol dehydrogenase RDH8 (6, 7) to all-*trans* retinol in a reaction that uses NADPH (8). All-*trans* retinol is transferred to the adjacent retinal pigment epithelium (RPE) cells, where it is converted to 11-*cis* retinal, which is then transported to rod photoreceptor outer segments, where it reforms rhodopsin (9, 10, 11). Apart from beginning the recycling of the photoisomerized chromophore, the reduction of all-*trans* retinal to all-*trans* retinol also accomplishes the removal of all-*trans* retinal, a highly reactive aldehyde and photosensitizer (12, 13) with a range of cytotoxic effects (14). Furthermore, all-*trans* retinal reacts with outer segment components to form *bis*-retinoid adducts (15), which enter the RPE cells through phagocytosis of the outer segments (16). Within the RPE, *bis*-retinoids undergo further processing and accumulate in the lysosomal compartment as part of the fluorescent pigment lipofuscin (17, 18). Lipofuscin, as well as its *bis*-retinoid components, displays a range of cytotoxic effects that include phototoxicity and inhibition of mitochondrial and lysosomal function (19, 20, 21, 22, 23).

Because of the toxicity of all-*trans* retinal, as well as that of its *bis*-retinoid adducts, defects in all-*trans* retinal removal have been proposed to play a role in the pathogenesis of retinal degeneration diseases such as Age-related Macular Degeneration (24, 25). A compromised photoreceptor metabolism would impair the removal of all-*trans* retinal and could contribute to the development of retinal degeneration. We have previously used fluorescence imaging of single isolated mouse rod photoreceptors to measure the levels of all-*trans* retinal, all-*trans* retinol, and lipofuscin fluorophore precursors (LFPs) in their outer segments, and used the extent of conversion of endogenously released all-*trans* retinal to all-*trans* retinol as a measure of their NADPH-generating capacity (26). We considered using the same approach for measuring the NADPH-generating capacity of human rod photoreceptors isolated from donor eyes, but these cells contain highly variable, often negligible, amounts of rhodopsin, and do not reliably generate sufficient levels of endogenous all-*trans* retinal. We circumvented this limitation by supplying isolated human rods with exogenous all-*trans* retinal and used the extent of its conversion to all-*trans* retinol as a measure of NADPH-generating capacity. Using exogenously supplied all-*trans* retinal, we measured the levels of all-*trans* retinal, all-*trans* retinol, and LFPs in the outer segments of isolated human rod photoreceptors with fluorescence imaging. We then examined the dependence of NADPH-generating capacity on extracellular glucose concentration and on time after donor death and isolation of the retina, and the effect of photoreceptor metabolic impairment on the formation of LFPs.

## Results

**Figure 1:**
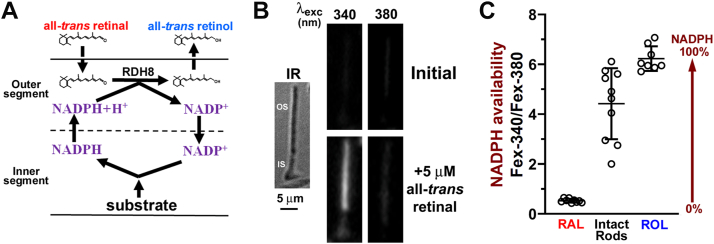
**Measurement of NADPH-generating capacity of isolated human rod photoreceptors from the conversion of exogenously supplied all-*trans* retinal to all-*trans* retinol.***A*, schematic diagram of the assay showing the use of the rod outer segment as a mini test tube where the reduction of all-*trans* retinal to retinol is monitored. NADPH is generated by the metabolic machinery in the rod inner segment and consumed in the outer segment in the reduction reaction catalyzed by retinol dehydrogenase RDH8. *B*, reduction of exogenous all-*trans* retinal, supplied extracellularly with bovine serum albumin, to all-*trans* retinol in a human intact rod photoreceptor; IR, bright field infrared image; OS, outer segment; IS, inner segment. Fluorescence images were acquired with 340 and 380 nm light excitation (emission >420 nm), before the addition (Initial), and 5 min after the addition of 5 μM all-*trans* retinal. *C*, Fex-340/Fex-380 ratio of the fluorescence excited by 340 and 380 nm (emission >420 nm) for intact rod cells after exposure to 5 μM all-*trans* retinal (n = 10, from two donors, ages 76 and 80 years). The fluorescence ratios for all-*trans* retinal (RAL) and all-*trans* retinol (ROL) are also shown, replotted from (28). Error bars represent mean ± SD. Open circles (◯) represent individual cell measurements. All experiments at 37 °C.

The outer segments of rod photoreceptors supplied with all-*trans* retinal reduce it to all-*trans* retinol using NADPH supplied by the metabolic machinery of the cell (Fig. 1*A*). Fig. 1*B* shows such an experiment with an isolated human rod photoreceptor. Outer segment fluorescence was excited with 340 and 380 nm light (emission >420 nm), which allows distinguishing the signals originating from all-*trans* retinal and all-*trans* retinol (27). Initial fluorescence images were recorded, then the cell was exposed for 5 min to 5 μM all-*trans* retinal delivered with 1% bovine serum albumin (BSA) as a lipophilic carrier, and fluorescence images were recorded again. The ratio Fex-340/Fex-380 of the fluorescence intensities excited by 340 (Fex-340) and 380 nm (Fex-380) reflects the fraction of all-*trans* retinol present, which is derived from the reduction of the supplied all-*trans* retinal. The value of the Fex-340/Fex-380 ratio determined from n = 10 cells was 4.42 ± 1.35 (mean ± SD) (Fig. 1*C*). The cells were isolated from two donors, aged 76 and 80 years, and were all metabolically intact, that is, the outer segments had attached inner segments, and thereby had access to the NADPH supply. The Fex-340/Fex-380 ratios for retinal (RAL, 0.53 ± 0.07, n = 9) and retinol (ROL, 6.23 ± 0.46, n = 8) are also shown, replotted from (28). From Equation 2, the value Fex-340/Fex-380 = 4.42 for the intact rod cells corresponds to a retinol fraction of 0.82.

**Figure 2:**
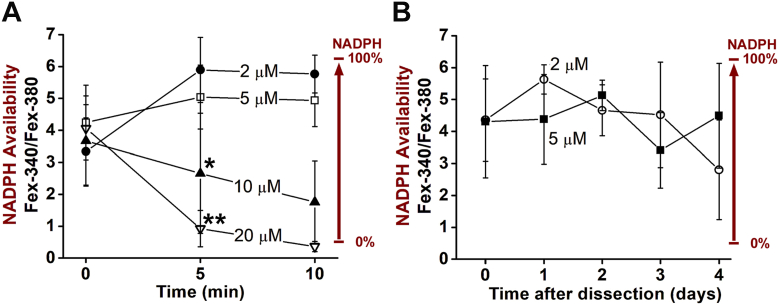
**Validation of the use of the concentration of 5 μM all-*trans* retinal for measuring the capacity of human rod photoreceptors to generate NADPH.***A*, values of the rod outer segment fluorescence ratio Fex-340/Fex-380 at different times after the addition of different concentrations of exogenous all-*trans* retinal, 2 (●, n = 11), 5 (□, n = 6), 10 (▲, n = 10), and 20 μM (▽, n = 8). Donor age, 74 years. At 5 min, the ratio elicited by 5 μM was not different from that elicited by 2 μM (two-tailed *t* test, *p* = 0.14), but was significantly higher than the ratios elicited by 10 and 20 μM (one-tailed t-tests, *p* = 0.01 and *p* < 0.001, respectively). The ratios elicited by 2 and 5 μM remain stable with time, they are not different at 10 compared to 5 min (two-tailed t-tests, *p* = 0.74 and 0.86, for 2 and 5 μM, respectively). Asterisks denote the level of statistical significance, *p* < 0.05 (∗) and *p* < 0.001 (∗∗). *B*, values of the outer segment fluorescence ratio Fex-340/Fex-380 elicited with 5 min exposures to 2 and 5 μM all-*trans* retinal from rods isolated at different days after the initial dissection of the retina. Cell numbers 4-8 for each determination; donor age, 86 years. The concentration used does not have a significant effect (two-factor ANOVA, *p* ≈ 1) on the value of the ratio. Error bars represent SD. Experiments at 37 °C.

The values of the Fex-380 and Fex-340 fluorescence intensities directly reflect the levels of all-*trans* retinal and all-*trans* retinol present, and virtually all of the Fex-340 fluorescence in particular originates from all-*trans* retinol (Equation 3 in (26)). Thus, they provide a measure of the all-*trans* retinal load on the enzymatic machinery of the rod cell. The value of the Fex-340 intensities obtained with exogenous all-*trans* retinal was 460 ± 188 f.a.u. (mean ± SD, n = 10), several times higher than the value obtained with endogenously generated all-*trans* retinal, 107 ± 41 f.a.u. (mean ± SD, n = 8, data from the cells in Fig. 2*D* in (28)).

A proper measurement of the capacity of the cell to generate NADPH and reduce all-*trans* retinal to all-*trans* retinol should provide values that are independent of the specific concentration of supplied all-*trans* retinal and exposure time. Therefore, we examined the effect of supplying all-*trans* retinal concentration and exposure time on the Fex-340/Fex-380 ratio in some detail (Fig. 2*A*). There were significant differences between the Fex-340/Fex-380 ratios elicited by different concentrations of all-*trans* retinal with a 5-min exposure time (one-factor ANOVA, *p* < 0.001). Compared to the ratio elicited by 5 μM all-*trans* retinal, the ratio elicited by 2 μM was not significantly different (two-tailed *t* test, *p* = 0.14), but the ratios elicited by 10 and 20 μM were significantly lower (one-tailed t-tests, *p* = 0.01 and *p* < 0.001, respectively). This indicates that with 10 and 20 μM the metabolic machinery of the cell cannot generate the levels of NADPH required for maintaining the reduction of the continuously supplied all-*trans* retinal, while the 5 μM concentration does not overwhelm the metabolic machinery of the cell. For 2 and 5 μM, the Fex-340/Fex-380 ratios remain stable with time; they are not significantly different at 10 min compared to 5 min after the addition (two-tailed t-tests, *p* = 0.74 and 0.86 for 2 and 5 μM, respectively). These results support the validity of the measurement of the NADPH-generating capacity of the cells using the particular combination of all-*trans* retinal concentration (5 μM) and exposure time (5 min). Additional support is provided by the similar value for the Fex-340/Fex-380 ratio, 4.42, measured from the reduction of 5 μM exogenous all-*trans* retinal and that measured from the reduction of endogenously released all-*trans* retinal ((28), see Discussion). Because the NADPH-generating capacity of cells declines with time after the initial dissection of the retina (see Fig. 4*A*), we also examined whether the supply of different concentrations of all-*trans* retinal would result in different values for the Fex-340/Fex-380 ratio at different times post-dissection. Figure 2*B* shows that exposures to 2 and 5 μM all-*trans* retinal resulted in the same values (two-factor ANOVA, *p* ≈ 1) for the Fex-340/Fex-380 ratio measured at different times after the initial dissection of the retina. This result further validates the use of 5 μM all-*trans* retinal for measuring the NADPH-generating capacity of the cells.

**Figure 3:**
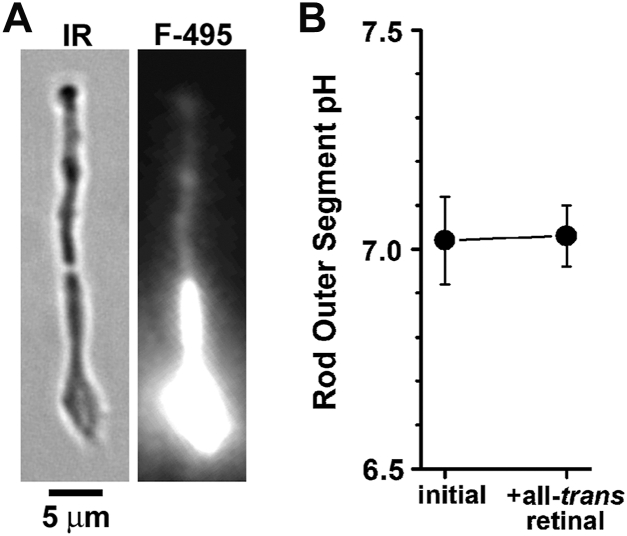
**Measurement of human rod photoreceptor outer segment pH with BCECF, a ratiometric pH-sensitive dye.** BCECF fluorescence was excited with 495 and 440 nm light, and emission was measured >515 nm. *A*, infrared bright field (IR) and fluorescence (495 nm excitation, F-495) images of an isolated human rod photoreceptor loaded with BCECF. The fluorescence image intensity is scaled so that the outer segment BCECF signal, which is used for the pH measurement, is readily visible. At this scaling, the inner segment signal—which is not used for the measurement—is saturated. *B*, outer segment pH of isolated human rod photoreceptors (n = 12) before and 5 min after the addition of 5 μM all-*trans* retinal. Donor age, 27 years. The addition of 5 μM all-*trans* retinal for measuring metabolic activity does not change human rod outer segment pH (two-tailed *t* test, *p* = 0.81). Error bars represent SD. Experiments at 37 °C.

Because H^+^ participates in the reduction of all-*trans* retinal to all-*trans* retinol (Equation 1), the value of the outer segment H^+^ concentration is needed to calculate the fraction of reduced NADP from the fraction of all-*trans* retinol. We used the fluorescent pH-sensitive dye BCECF to measure outer segment pH with single-cell fluorescence imaging (Fig. 3). The initial rod outer segment pH value was 7.02 ± 0.10, and did not change (two-tailed *t* test, *p* = 0.81) with the addition of 5 μM all-*trans* retinal for 5 min, the value being 7.03 ± 0.07 (n = 12 cells, donor age 27 years). With a value of 7.0 for the outer segment pH, the Fex-340/Fex-380 ratio value of 4.42 corresponds to a reduced NADP fraction of 0.14 (Equation 3).

**Figure 4:**
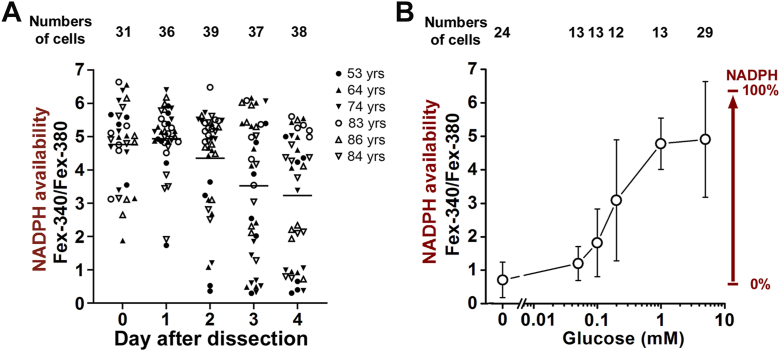
**Dependence of NADPH-generating capacity of isolated human rod photoreceptors on the time after the initial dissection of the retina and on glucose availability.***A*, the NADPH-generating capacity of isolated human rods declines with time after dissection (linear regression, slope = −0.46 ± 0.09, *p* < 0.001) but is stable for the first 3 days (one-factor ANOVA, *p* = 0.13). Cells were isolated from 6 pairs of donor eyes, with donor ages 53 to 86 years. Measurements at 37 °C in the presence of 5 mM glucose. Bars represent the grand mean from all cells on each day. *B*, NADPH-generating capacity of isolated human rods depends on the availability of extracellular glucose. Cells were isolated from 5 pairs of donor eyes, with donor ages 74 to 84 years. Measurements at 37 °C in the presence of different concentrations of glucose. Error bars represent SD.

**Figure 5:**
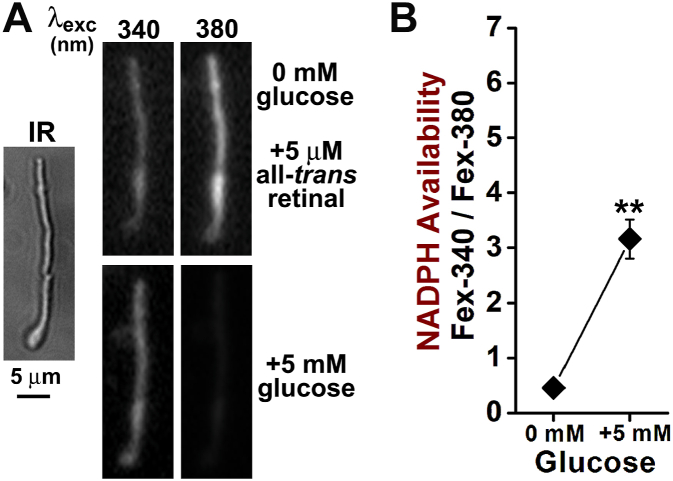
**Incubation in the absence of metabolic substrate does not permanently impair the capacity of human rod photoreceptors to generate NADPH.***A*, incubation of an isolated human rod photoreceptor with all-*trans* retinal in the absence of glucose results in the accumulation of all-*trans* retinal in the outer segment. Subsequent addition of 5 mM glucose results in a reduction of the accumulated all-*trans* retinal to all-*trans* retinol. IR, bright field infrared image; fluorescence images shown were acquired with 340 and 380 nm light excitation (emission >420 nm) after incubation for 5 min with 5 μM all-*trans* retinal without glucose, and then 5 min after the addition of 5 mM glucose. *B*, Fex-340/Fex-380 fluorescence ratio at the end of the 5 min incubation with 5 μM all-*trans* retinal without glucose and following the addition of 5 mM glucose (n = 7, donor age 86 years). The ratio increases (one-tailed *t* test, *p* < 0.001) from a value of 0.45 ± 0.02 (mean ± SD), indicative of all-*trans* retinal, to a value of 3.16 ± 0.15, demonstrating the conversion to all-*trans* retinol. Asterisks (∗∗) represent statistical significance *p* < 0.001. Error bars represent SD. Experiments at 37 °C.

After the initial dissection of the eyes and isolation of the retinas, the retinas were kept in physiological solution at 4 °C, and when there was time for an experiment, rod photoreceptors were isolated from a piece of retina. It was evident that the health of the rod photoreceptors deteriorated with time after the initial dissection, as more cells appeared swollen after isolation, and it was more difficult to find intact cells for an experiment. This deterioration in health was manifested in the NADPH-generating capacity of the cells (Fig. 4*A*), as reflected in the decline of the Fex-340/Fex-380 ratio with time after the initial dissection (linear regression, slope = −0.46 ± 0.09, *p* < 0.001). We used regression to test whether there was a significant decline in NADPH-generating capacity with time after the initial dissection, but there is no expectation that the decline is linear with time. Indeed, the decline was not linear, and no difference was detected (one-factor ANOVA, *p* = 0.13) in the NADPH-generating capacity for the first 3 days. For these experiments, cells isolated from 6 pairs of donor eyes (donor ages 53–86 years) were used, with at least 4 cells used from each donor for each day. The ability of the cells to generate NADPH required the presence of glucose. In the absence of glucose, the Fex-340/Fex-380 ratio was 0.70 ± 0.53 (mean ± SD, n = 24), not significantly different from the value 0.53 ± 0.07 (n = 9) for all-*trans* retinal (two-tailed *t* test, *p* = 0.35), that is, there was no detectable reduction of all-*trans* retinal. The NADPH-generating capacity depended on the extracellular concentration of glucose (Fig. 4*B*) and was compromised below 0.2 mM. The inability of the cells to reduce the supplied all-*trans* retinal in the absence of glucose was due to the lack of metabolic substrate and not to impairment of the metabolic machinery: supplying glucose to cells pre-loaded with all-*trans* retinal in the absence of glucose resulted in reduction of the accumulated all-*trans* retinal (Fig. 5).

**Figure 6:**
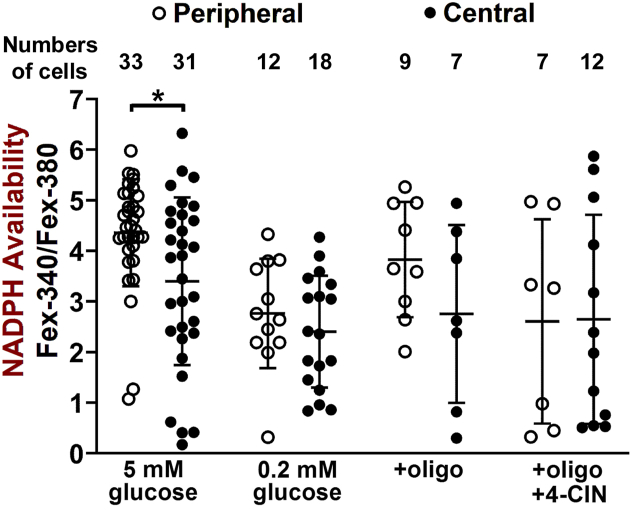
**Comparison of the NADPH-generating capacity of human rod photoreceptors isolated from central and peripheral areas of the retina.** Error bars represent mean ± SD. In the presence of 5 mM glucose, NADPH-generating capacity of human rods isolated from the central retina is lower than that from the peripheral retina (one-tailed *t* test, *p* = 0.004). The NADPH generating capacity of rods isolated from central and peripheral retina are not significantly different in the presence of 5 μM oligomycin (+oligo, two-tailed *t* test, *p* = 0.16), in the presence of 5 μM oligomycin and 1 mM 4-CIN (+oligo+4-CIN, two-tailed *t* test, *p* = 0.97), or in the presence of 0.2 mM glucose (two-tailed *t* test, *p* = 0.38). Asterisk denotes the level of statistical significance, *p* < 0.05 (∗). Cells were isolated from 5 pairs of donor eyes, with donor ages 59 to 83 years. All experiments at 37 °C.

For experiments, we typically isolated rod cells from the periphery, and specifically from the ring surrounding the macula, where their density is highest (29). Because of the importance of the central area of the retina for vision, we examined whether there were differences in the NADPH-generating capacity of rods isolated from the central compared to the peripheral areas. The results of Figure 6 show that in the presence of 5 mM glucose, the NADPH-generating capacity of central rods was significantly lower than that of peripheral (one-tailed *t* test, *p* = 0.004). We further compared the NADPH-generating capacities of central and peripheral rods under metabolic conditions that decreased the amounts of metabolic substrate available for the generation of NADPH. These conditions included a reduced extracellular glucose concentration (0.2 mM), inhibition of the ATP-synthase with oligomycin, and inhibition of mitochondrial metabolic contributions with oligomycin and the pyruvate transporter inhibitor α-cyano-4-hydroxy-cinnamate (4-CIN) (27). The experiments with oligomycin and 4-CIN were carried out in the presence of 5 mM glucose. No significant differences were detected in the NADPH-generating capacities of central and peripheral rods under any of the three conditions (Fig. 6). Unfortunately, it was not possible to compare central and peripheral rod metabolism with glutamine as metabolic substrate, because BSA, the carrier for the exogenously supplied all-*trans* retinal, would interfere with glutamine utilization by the cell (30).

**Figure 7:**
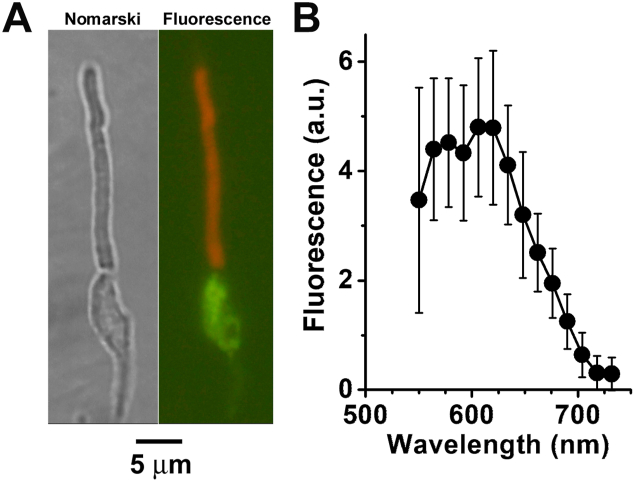
**Lipofuscin fluorophore precursors in the outer segments of isolated human rod photoreceptors.***A*, Nomarski and true color image of the fluorescence (excitation: 450–490 nm) emitted by an isolated human rod photoreceptor (donor age 80 years). The inner segment fluorescence (*green color*) is likely due to mitochondrial FAD. *B*, Emission spectra of human rod outer segment fluorescence excited by 488 nm light (n = 17, donor age 79 years). Error bars represent SD.

**Figure 8:**
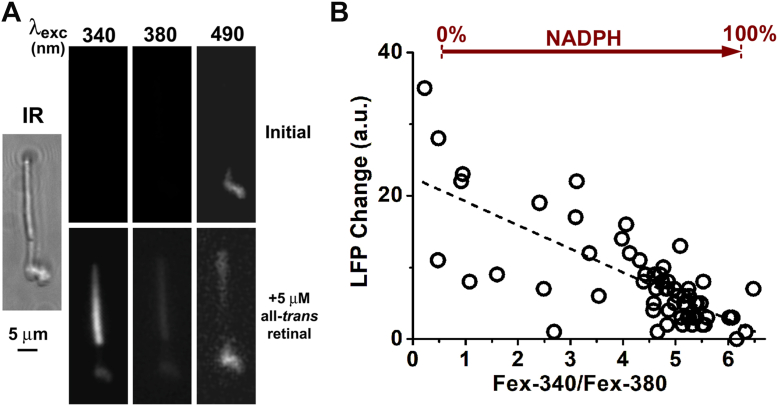
**Decreased reduction of all-*trans* retinal by human rod photoreceptors results in increased formation of lipofuscin fluorophore precursors (LFP) in outer segments.***A*, conversion of exogenously supplied all-*trans* retinal to all-*trans* retinol and formation of LFP in an isolated human rod photoreceptor (donor age 83 years). IR, infrared image of the cell. Fluorescence images were acquired before and 5 min after the addition of 5 μM all-*trans* retinal. Fluorescence images acquired with 340 and 380 nm light excitation (emission >420 nm) were used to measure the conversion of all-*trans* retinal to all-*trans* retinol, and with 490 nm excitation (emission >515 nm) to measure LFP formation. *B*, in isolated human rod photoreceptors (n = 62, two donors, ages 64 and 83 years), lower conversion of exogenously supplied all-*trans* retinal to all-*trans* retinol results in increased LFP formation (linear regression, slope = −3.32 ± 0.38, *p* < 0.001, dashed line). Fluorescence intensities were measured as in *panel A*, before and 5 min after the addition of 5 μM all-*trans* retinal, and the change in intensities was used to calculate the Fex-340/Fex-380 ratio and the increase in LFP levels. In the graph, the value Fex-340/Fex-380 = 0.53 corresponds to 100% retinal-0% retinol and a reduced NADP fraction of 0%; Fex-340/Fex-380 = 6.23 corresponds to 0% retinal-100% retinol and a reduced NADP fraction of 100%. To obtain cells with low Fex-340/Fex-380 ratios, measurements were carried out beyond the first 3 days after the initial dissection of the retina. All experiments at 37 °C in the presence of 5 mM glucose.

The outer segments of isolated human rod photoreceptors emitted an orange fluorescence when excited by blue light (Fig. 7*A*), with an emission spectrum peak of ∼600 nm (Fig. 7*B*), similar to that emitted by RPE lipofuscin granules (31). We have been referring to the fluorophores responsible for this fluorescence as lipofuscin fluorophore precursors (LFPs), and we have previously shown that they originate from reactions of 11-*cis* and all-*trans* retinal with outer segment components (31). Here we examined whether the exogenously supplied all-*trans* retinal would form LFPs and whether their levels would depend on the extent of the reduction of all-*trans* retinal to all-*trans* retinol. Figure 8*A* shows that addition of exogenous all-*trans* retinal to human rods does increase the levels of LFPs in the outer segment. Moreover, the increase in LFP levels depended on the extent of reduction of the supplied all-*trans* retinal, and lower Fex-340/Fex-380 fluorescence ratios correlated with larger increases in LFP levels (Fig. 8*B*, linear regression, slope = −3.32 ± 0.38, *p* < 0.001). For Figure 8*B*, we carried out experiments with cells isolated on days 0, 1, and 2 after the initial dissection, when cells maintained robust reduction of supplied all-*trans* retinal (Fig. 4*A*), but we also carried out experiments on days 3 and 4, in order to obtain cells that were less effective at reducing all-*trans* retinal (Fig. 4*A*). In this way, we obtained cells with a wide range of Fex-340/Fex-380 fluorescence ratio values and LFP levels. It is important to note that the initial LFP levels, before supplying exogenous all-*trans* retinal, did not show any dependence on the metabolic capacity of the isolated cell as reflected in the similar Fex-340/Fex-380 ratios (linear regression, *p* = 0.18; data not shown).

## Discussion

The death of photoreceptor cells is one of the major causes of vision loss. In normal human eyes, rod photoreceptors are selectively lost with age in the central retina (32), a loss significantly accelerated in eyes afflicted with Age-related Macular Degeneration (AMD) (33), one of the leading causes of blindness in industrialized countries (34). Photoreceptor cell death is also the cause of vision loss in inherited retinal degenerations, diseases that are characterized by progressive dysfunction and death of photoreceptor cells.

A cell needs to be metabolically competent in order to meet the energy demands of its physiological functions and maintain its viability. A compromised metabolism can result in loss of cellular function and ultimately death. We have examined the metabolic health of human rod photoreceptors through their capacity to generate NADPH by measuring the conversion of exogenously supplied all-*trans* retinal to all-*trans* retinol. For our measurements, we exposed a cell to 5 μM all-*trans* retinal for 5 min (Fig. 1), so we ascertained that this exposure did not overload the metabolic machinery of the cell (Fig. 2). First, a lower concentration, 2 μM, of all-*trans* retinal resulted in the same fraction converted to all-*trans* retinol, and this fraction remained stable for an exposure up to 10 min. Second, as the metabolic health of the cells deteriorated with time after death, they converted the same fraction of all-*trans* retinal to all-*trans* retinol in the presence of either 2 or 5 μM.

When exposed to 5 μM all-*trans* retinal with 5 mM glucose as metabolic substrate, the cells maintained a fraction of all-*trans* retinol of ∼0.8 to 0.9, as reflected in the Fex-340/Fex-380 ratio of 4.42. This is similar to the fraction of endogenously released all-*trans* retinal converted to all-*trans* retinol (28), but we further observed that the level of outer segment fluorescence generated from exogenous all-*trans* retinal is several times higher than that from endogenous. This suggests that the NADPH-generating capacity of rod photoreceptor cells is more than adequate to support the reduction of the all-*trans* retinal released by photoactivated rhodopsin following light exposure, that is, the supply of NADPH is not limiting. As evidenced by the conversion of lower proportions of all-*trans* retinal to all-*trans* retinol, the supply of NADPH appears to become limiting at 10 and 20 μM all-*trans* retinal concentrations (Fig. 2*A*), which however, represent loads several times higher than the physiologically encountered. The cells are, of course, generating NADPH in a continuous fashion, but the supply of all-*trans* retinal is also continuous. A limited ability of the cell to generate NADPH would provide an explanation for the lower fraction of retinal converted to retinol, as at the higher retinal concentrations the generation of NADPH would not be able to keep up with the supply. Other explanations for the lower conversion are also possible, for example, the higher concentrations of all-*trans* retinal could inhibit the retinol dehydrogenase enzyme. It should be noted that the capacity of rod photoreceptors to generate NADPH does not appear to decline with donor age, as most of the results shown in Figures 1*C* and 4*A* were from donors older than 70 years, with several being older than 80 years.

In addition to NADPH, the reduction of all-*trans* retinal to all-*trans* retinol consumes H^+^. The rod outer segment pH of ∼7.0 is not affected by the exposure to 5 μM all-*trans* retinal for 5 min (Fig. 3), which suggests strong H^+^ buffering within the compartment. The stable value of the H^+^ concentration during the course of the experiment also allowed the straightforward calculation of the reduced fraction of NADP. The value of 0.14 for the reduced fraction of NADP corresponds to a [NADPH]/[NADP^+^] ratio of 0.16, in broad agreement with histochemical measurements (35). The pH value of ∼7.0 for human rod outer segments is similar to the 7.0 to 7.1 value for bleached mouse rods (27), consistent with the bleached state of the human cells used for the experiments herein.

A major concern with the assessment of the metabolic capacity of the isolated rod photoreceptor cells is its impairment due to the long period of post-mortem ischemia preceding the isolation of the retinas. A related concern is that this period of post-mortem ischemia differed for eyes from different donors, thereby increasing the potential variability. Nevertheless, for the first 3 days after the initial dissection of the globes and isolation of the retinas, isolated rod cells obtained from different donors exhibited robust metabolic capacity, demonstrated by their ability to maintain reduction of 80 to 90% of the supplied all-*trans* retinal to all-*trans* retinol (Fig. 4*A*). This ability indicates the lack of any impairment of the NADPH-generating machinery, as it is indistinguishable from that exhibited by mouse rod cells isolated from freshly dissected retinas subjected to no significant period of ischemia (27). We have therefore used cells isolated during the first 3 days for examining the dependence of NADPH-generating capacity on different factors. Given the lack of any detectable metabolic impairment during the first 3 days after globe dissection, we did not attempt to analyze the potential effect of any relatively minor differences in procurement parameters (the times after death to globe preservation and to retina isolation) across donors. NADPH-generating capacity did decline in later days (Fig. 4*A*), and we used cells isolated in those days to examine the effects of metabolic impairment on the formation of LFPs (Fig. 8).

Reducing the extracellular glucose concentration decreased NADPH-generating capacity (Fig. 4*B*). As these experiments were carried out with cells isolated in the first 3 days after isolation of the retinas, this decrease was not due to impairment in metabolic machinery, but rather reflected the need for a continuous supply of metabolic substrate to generate NADPH. In the absence of glucose, there was no detectable conversion of all-*trans* retinal to all-*trans* retinol, indicating the lack of any metabolite pools that could provide significant amounts of metabolic substrate perhaps *via* catabolic reactions. The dependence of NADPH-generating capacity on glucose concentration is broadly similar to that for mouse rod cells—measured from the reduction of endogenously released all-*trans* retinal—where NADPH generation is compromised below ∼0.1 to 0.2 mM glucose (27). Interestingly, in mouse rods, there is some reduction in all-*trans* retinal to all-*trans* retinol even in the absence of any extracellular glucose, suggesting the intracellular availability of low levels of metabolic substrate. In human rods, the lack of significant intracellular metabolite pools may be the result of depletion due to the prolonged ischemia and the high exogenous all-*trans* retinal loads. It is important to note that the absence of glucose did not result in the irreversible impairment of metabolic capacity, as upon resupplying glucose, the cells were able to reduce all-*trans* retinal (Fig. 5).

The lack of metabolic impairment due to the short-term absence of glucose or to the prolonged ischemia before isolation of the retinas suggests a remarkable resilience of human rod photoreceptors to ischemic insult, in marked contrast to inner retina neurons that are known to be far more susceptible (36). The number of rod photoreceptors in the central retina however, has been known to decrease with age, and this decrease is significantly accelerated in Age-related Macular Degeneration (32, 33). To probe whether there is a potential metabolic basis for this decrease that occurs even in otherwise healthy eyes, we examined whether there were any differences between central and peripheral rod photoreceptors in their ability to generate NADPH. The NADPH-generating capacity of rods isolated from the central retina was significantly lower than that of rods isolated from the peripheral (Fig. 6). However, no significant differences were found when the availability of metabolic substrate for the generation of NADPH was decreased in the following ways: (i) a lower glucose concentration (0.2 mM), which would directly reduce the metabolic substrate available for NADPH generation (Fig. 4*B*), (ii) exposure to oligomycin, an ATP synthase inhibitor, which would draw off metabolic substrate from NADPH generation to be used for ATP synthesis *via* glycolysis (27), and (iii) exposure to a combination of oligomycin and 4-CIN (a pyruvate transporter inhibitor), which would be expected to shift ATP and NADPH production wholly onto glycolysis and the pentose phosphate pathway (27). In view of the lower NADPH-generating capacity of central rods in the presence of 5 mM glucose, the failure to find significant differences under conditions that reduce the metabolic substrate available for NADPH generation is surprising. A plausible explanation is that there are no intrinsic metabolic differences between central and peripheral rods, but the central retina is more sensitive to the prolonged period of ischemia. This sensitivity would be consistent with the challenge of isolating viable rod photoreceptors from the central human retina, a difficulty we encountered with human cone photoreceptor isolation as well, while isolating cones from the central retina of monkey eyes subjected to only a few hours of ischemia presented much less of a challenge (28). Such sensitivity could result in the metabolic impairment of central rods. This metabolic impairment of central rods compared to peripheral would be masked under the conditions of decreased substrate availability due to the additional metabolic stress. The central retina is expected to have higher metabolic requirements than the peripheral retina due to the higher concentration of cones (37). Under ischemic conditions, the higher metabolic activity would result in faster depletion of metabolite pools and make the central retina more sensitive to the post-mortem ischemic insult compared to the peripheral. Overall, the results are consistent with a higher susceptibility of the central retina to metabolic stress, which may be an underlying factor in the decrease in the numbers of rods in this area with age.

The true color and emission spectrum of the human rod outer segment fluorescence excited by blue light (Fig. 7) are similar to those measured in mouse rods (31). In the case of mouse rods, the lipofuscin fluorophore precursors (LFPs) responsible for this fluorescence can originate from reactions of either all-*trans* or 11-*cis* retinal with outer segment components (31). They are also detected in the rod outer segments of dark-reared animals in the absence of any prior light exposure, hence their original source has been suggested to be 11-*cis* retinal (31). In the case of human rods, which have been isolated from donor eyes that have been exposed to light and subjected to prolonged ischemia, it is possible that all-*trans* retinal has contributed significantly to the formation of the detected fluorophores. All-*trans* retinal can clearly promote LFP formation (Fig. 8), with LFP levels increasing with less reduction of all-*trans* retinal to all-*trans* retinol, which would correspond to decreased NADPH availability. The same relationship between all-*trans* retinal reduction and LFP formation has been found in mouse rods, using the endogenously generated all-*trans* retinal (26). Previous work has shown that increases in lipofuscin precursor formation are the result of defects in enzymes involved in all-*trans* retinal clearance, such as ABCA4 (38), RDH8 (7), or RDH12 (39). The current work demonstrates that in human rod photoreceptors, a metabolic impairment would also increase the formation of lipofuscin precursors. This inverse correlation between LFP formation and NADPH availability points to the possible operation of a positive feedback loop involving the photoreceptors and the adjacent RPE cells. This feedback loop would reinforce declines in photoreceptor metabolic health, as an initial deterioration would be accompanied by a decline in NADPH-generating capacity, which would result in increased LFP formation in outer segments. LFPs would then enter the adjacent retinal pigment epithelial cells through the phagocytosis of the outer segments and would accumulate in the lysosomes in the form of lipofuscin. The health of the RPE cells would decline due to the increased lipofuscin load, diminishing their support of the adjacent photoreceptors, leading to further deterioration of their health and eventually result in cell death. Such a feedback loop suggests yet another mechanism through which defects in the clearance of all-*trans* retinal (24, 25) could contribute to the development of retinal degeneration. The operation of such a positive feedback loop would be local and would be expected to manifest itself as a focal increase in RPE lipofuscin that over time would progress to a lesion encompassing both RPE and photoreceptors. One potential test of the operation of this mechanism would be *in vivo* fluorescence imaging of human retinal pigment epithelium with adaptive optics (40), which could detect focal increases of lipofuscin fluorescence and monitor their progression in time. Another approach would be MALDI Imaging Mass Spectrometry of human cadaver eyes to examine the relation between the focal accumulation of *bis*-retinoids such as A2E and disease-associated lesions (41, 42).

In summary, we have used the extent of reduction of all-*trans* retinal to all-*trans* retinol to characterize the capacity of single living isolated human rod photoreceptors to generate NADPH. We find that the generation of NADPH is dependent on the availability of extracellular glucose as a metabolic substrate. The cells are able to generate NADPH at rates several times higher than those that would be required to fulfill physiological demands, and exhibit a remarkable resilience to prolonged ischemia. Rods isolated from the central retina have lower NADPH-generating capacity compared to those from the periphery, which might be due to a higher susceptibility of the central retina to prolonged ischemia. Finally, all-*trans* retinal forms lipofuscin fluorophore precursors, whose levels increase when the extent of all-*trans* retinal reduction decreases. This could underlie the operation of a positive feedback loop, through which modest initial decreases in photoreceptor metabolism would promote the increased accumulation of lipofuscin in the adjacent RPE cells, leading to impairment of their function followed by further decreases in photoreceptor metabolism, and progressing eventually to cell death.

## Experimental procedures

### Human donor eyes

Eyes, from donors without diagnosed eye disease or diabetes mellitus, were procured through National Disease Research Interchange (NDRI). We followed our previously published protocol (28): eyes were enucleated within 10 h *post mortem*, placed in moist chambers, and then shipped to the laboratory on ice in a light-tight container. Retinas were isolated from the eyes within 48 h after donor death; upon inspection, the retinas did not show any signs of pathology. Eyes from 18 donors, male and female, ages 27 to 86 years, were used in this study.

### Isolation of single rod photoreceptor cells

Retinas were excised under either dim red or infrared light in mammalian physiological solution (mmol/L: 130 NaCl, 5 KCl, 0.5 MgCl_2_, 2 CaCl_2_, 25 hemisodium-HEPES, 5 glucose, pH = 7.40). With the exception of the measurements of the rod outer segment emission spectra (Fig. 7), the retinas were bleached with white light to remove any residual rhodopsin present. Retinas were kept in a light-tight container at 4 °C until used for the isolation of cells. The macula was identified as described (28), and for most experiments, rod photoreceptors were isolated from the rod-rich area surrounding the macula. For experiments with rod photoreceptors isolated from the central area (Fig. 6), a 7.25 mm diameter punch of the area surrounding the fovea was obtained with a trephine blade. Single photoreceptor cells were obtained by chopping a piece of retina with a razor blade as described (43). Unless explicitly stated otherwise, the photoreceptors used for experiments were isolated within the first 3 days after dissection of the retina (referred to as days 0, 1, and 2). Experiments with cells isolated on later days (days 3 and 4) were carried out to characterize declines in metabolic capacity (Figure 2, Figure 4*B* and 4*A*), or the effect of the decline in metabolic capacity on the formation of lipofuscin fluorophore precursors (Fig. 8).

### Fluorescence imaging

Fluorescence imaging experiments were carried out on the stage of an inverted Zeiss Axiovert 100 microscope (Carl Zeiss, Thornwood, NY) with a 40 × oil immersion objective lens (N.A. = 1.3) as described previously (43). For a typical experiment, initial fluorescence images were recorded, then 5 μM all-*trans* retinal was added with 1% bovine serum albumin (BSA) as a carrier, and fluorescence images were recorded again after 5 min. For some experiments, 2, 10 or 20 μM concentrations of all-*trans* retinal were used, while in others, images were recorded immediately and 10 min after the addition of all-*trans* retinal. Fluorescence intensity was measured over defined regions of interest (ROI) in the outer segments and background. To quantify the capacity of a rod cell to generate NADPH (27), the intensities of the fluorescence excited by 340 and 380 nm light with emission collected >420 nm were measured. After background correction, the initial outer segment fluorescence intensities were subtracted from the values measured at 5 min (or 0 and 10 min for some experiments) after the addition of all-*trans* retinal and the values Fex-340 and Fex-380 of the increase in fluorescence intensities due to the added all-*trans* retinal were obtained. The ratio Fex-340/Fex-380 represents the capacity of the cell to generate NADPH. For lipofuscin fluorophore precursor (LFP) measurements, fluorescence was excited with 490 nm light and emission collected >515 nm (31). The intracellular pH of outer segments was measured with BCECF, a ratiometric pH-sensitive dye, according to procedures previously used with isolated salamander and mouse rod photoreceptors (27, 44). BCECF fluorescence was excited with 495 and 440 nm light, emission was measured >515 nm, and the ratio F_495_/F_440_ of the fluorescence intensities excited by 495 nm (F_495_) and 440 nm (F_440_) was converted to pH using a calibration curve (27). Image acquisition and analysis were carried out using Slidebook (Intelligent Imaging Innovations, Denver, CO).

Fluorescence emission spectra of outer segments of isolated rods were measured with an SP2 Leica Laser Scanning Confocal Microscope, at room temperature, with a 63 × oil immersion lens (NA = 1.4) using 488 nm excitation as described previously (31). Spectra were corrected for background by subtracting the fluorescence intensities of the spectrum of an area from the same field that did not contain cells or tissue debris.

Color photographs were taken on a Zeiss Axioplan 2 microscope (Carl Zeiss) using a 63 × oil immersion objective (NA = 1.4) with a Nikon D200 (Nikon, Inc) digital camera. Fluorescence was excited with 450 to 490 nm light, and the emission was collected >510 nm.

### The Fex-340/Fex-380 ratio as a measure of NADPH availability in isolated rods

The reduction of all-*trans* retinal to all-*trans* retinol in rod outer segments is coupled to the oxidation of NADPH to NADP^+^, in a reaction that also involves H^+^,

(Eq. 1) $$\text{retinal}+\text{NADPH}+H^{+}\;↔\;\text{retinol}+{\;\text{NADP}}^{+}$$

The availability of NADPH can be estimated in terms of the reduced fraction N_red_ = [NADPH]/([NADP^+^] + [NADPH]), which, from Equation 1, is directly related to the fraction [ROL]/([RAL] + [ROL]) (Equation 3 in (27)). The fraction [ROL]/([RAL] + [ROL]) can be obtained from the fluorescence intensity ratio FR = Fex-340/Fex-380, according to Eq. Five in (27), revised to take into account the slightly different Fex-340/Fex-380 ratios for [ROL] and [RAL] in human rods (6.23 and 0.53, respectively) compared to mouse (6.95 and 0.55, respectively).

(Eq. 2) [ROL]/([RAL]+[ROL])=(1.9×FR–1)/((1.9×FR–1)+QYR×(1–0.16×FR))

with 1.9 = 1/0.53, 0.16 = 1/6.23, and QYR is the ratio of the fluorescence quantum yield of all-*trans* retinol over that of all-*trans* retinal, which has been measured previously (6). The relationship between N_red_ and FR = Fex-340/Fex-380 is then given by (Eq. Six in (27), revised for human rods)

(Eq. 3) $$N_{\text{red}}=\left(1.9\times \text{FR}–1\right)\;/\;\left(\left(1.9\times \text{FR}–1\right)+\left(\left[H^{+}\right]/K_{\text{eq}}\right)\times \text{QYR}\times \left(1–0.16\times \text{FR}\right)\right)$$

K_eq_ = 3.3 × 10^−9^ M is the equilibrium constant for the reduction of all-*trans* retinal to all-*trans* retinol (45), and [H^+^] is the proton concentration in the human rod outer segment, corresponding to pH ∼ 7.0 (Fig. 3). We have used a value of 5.1 for QYR (6). We have therefore used the ratio of fluorescence intensities Fex-340/Fex-380 as a measure of NADPH availability.

### Materials

All reagents were of analytical grade. All-*trans* retinal, all-*trans* retinol, and lipid-free bovine serum albumin were from Sigma-Aldrich. The cell-permeant form of BCECF (the acetoxymethyl ester) was from Invitrogen.

### Statistical analysis

We used one- and two-factor ANOVA (with replication) to examine the effects of different variables. None of the ANOVA tests showed a significant effect of any of the variables tested, and no subsequent tests were carried out in those cases. To test the difference between means, we used one- and two-tailed *t*-tests. We used linear regression to test whether NADPH-generating capacity declined with time after the initial dissection and whether the formation of lipofuscin fluorophore precursors increased with decreases in NADPH-generating capacity. Results were considered statistically significant when *p* < 0.05.

## Data availability

Any data not contained within the article will be shared upon request. Contact Dr Yiannis Koutalos at koutalo@musc.edu.

## Conflict of interest

The authors declare that they have no conflicts of interest with the contents of this article.
